# Micro- and Nanoplastics as Environmental Stressors: Mechanistic Links Among Gut Dysbiosis, Inflammation, and Systemic Health Effects

**DOI:** 10.3390/jox16050161

**Published:** 2026-08-26

**Authors:** Guilherme de Oliveira Ferreira, Maria Júlia Ferreira Alves, Priscila Oliveira de Paula Charret, Thaysi Rodrigues, Natália A. Borges, Ludmila F. M. F. Cardozo, Denise Mafra

**Affiliations:** 1 Graduate Program in Nutrition Sciences, Fluminense Federal University (UFF), Niterói 24033-900, RJ, Brazil; guilhermeof@id.uff.br (G.d.O.F.); mariajuliaalves@id.uff.br (M.J.F.A.); pricharret@id.uff.br (P.O.d.P.C.); thaysirpa@id.uff.br (T.R.); ludmilacardozo@id.uff.br (L.F.M.F.C.); 2 Graduate Program in Food, Nutrition and Health—Institute of Nutrition, State University of Rio de Janeiro (UERJ), Rio de Janeiro 20550-900, RJ, Brazil; natalia.borges@uerj.br; 3 Graduate Program in Medical Sciences, Fluminense Federal University (UFF), Niterói 24033-900, RJ, Brazil; 4 Graduate Program in Biological Sciences—Physiology, Federal University of Rio de Janeiro (UFRJ), Rio de Janeiro 21941-941, RJ, Brazil

**Keywords:** microplastics, nanoplastics, gut microbiota, inflammation, oxidative stress, chronic disease, environmental exposure

## Abstract

Microplastics and nanoplastics (MNPs) have emerged as widespread environmental contaminants, with increasing evidence suggesting that chronic exposure may adversely affect human health. Experimental and emerging human studies indicate that MNPs can interact with multiple biological systems and trigger a range of mechanisms, including oxidative stress, mitochondrial dysfunction, cellular injury, barrier disruption, and immune and inflammatory responses. These effects may involve direct interactions with tissues and cells as well as indirect pathways, including alterations in gut microbial and intestinal homeostasis. In turn, persistent inflammatory and metabolic disturbances may contribute to tissue dysfunction and the development or progression of chronic diseases. This narrative review summarizes current evidence on the biological effects of MNP exposure, focusing on the mechanisms linking environmental exposure to chronic inflammation and disease. We discuss their potential involvement in cardiovascular, liver, kidney, respiratory, and neurological diseases, while highlighting the emerging role of the gut microbiota and intestinal barrier as potential modulators of these effects. Given the limitations of current human evidence and the heterogeneity of experimental models, we also discuss major knowledge gaps and the need for further mechanistic and epidemiological studies to clarify the clinical relevance of chronic MNPs exposure.

## 1. Introduction

Few inventions have transformed human civilization as profoundly as plastics. From their discovery in the late 19th century to their widespread industrial use after 1907, plastics have revolutionized manufacturing, medicine, and daily life because of their versatility, durability, low cost, and accessibility [1]. Almost half of all plastics ever created have been produced in the last 15 years. It grew from about 2 million tons in 1950 to 368 million tons in 2019, an increase of almost 200 times. It is estimated that, by 2050, global production will double or triple, reaching between 900 and 1100 million tons [2,3].

Most plastics are derived from petrochemical sources, such as crude oil, natural gas, and coal, and account for approximately 4% of global fossil fuel production. They are synthetic polymers produced by polymerizing monomers, primarily composed of carbon and hydrogen, derived from petrochemicals such as ethylene and propylene. During manufacturing, various additives, including stabilizers, pigments, and flame retardants, are added to increase flexibility, durability, and performance [4,5,6].

The same versatility that once symbolized progress has become a global environmental challenge [1]. When released into the environment, plastic debris undergoes mechanical abrasion, thermal degradation, and exposure to ultraviolet (UV) radiation, forming small particles called microplastics (MPs) or nanoplastics (NPs) that vary in color, size, and shape. MPs are highly persistent contaminants that can accumulate in various environmental matrices, including soil, sediments, air, and aquatic ecosystems. Furthermore, in food chains, they can pose risks to ecosystem integrity and human health, as has already been detected in air, food, and drinking water [6,7].

Since the gut microbiota is highly sensitive to dietary and environmental factors, any ingested or inhaled substance, including MNPs, can affect its composition and function. This dysbiosis can disrupt host–microbe interactions, promoting intestinal inflammation and contributing to the development and progression of chronic diseases.

This narrative review examines the emerging interactions among MNPs, the gut microbiota, and inflammation, highlighting the underlying biological pathways, clinical consequences, and current research gaps. We further discuss how these interactions may contribute to the emergence and progression of chronic diseases. A deeper understanding of these interconnected mechanisms may pave the way for new preventive and therapeutic strategies to mitigate the potential health risks associated with exposure to MNPs.

## 2. Human Exposure and Biodistribution of MNPs

The accumulation and improper disposal of plastic waste in the environment have become major global concerns, as they lead to the formation of micro- and nanoplastics (MNPs) that can affect ecosystems and human health [8]. These polymers can form plastic particles that are generally classified as MPs when they are ≤5 mm in size, whereas particles smaller than 1 μm are typically classified as nanoplastics (NPs) [9]. Among the different types of plastic, the most widely used are polyethylene (PE) (high and low density, HDPE and LDPE), followed by polyvinyl chloride (PVC), polypropylene (PP), polyethylene terephthalate (PET), polystyrene (PS), and polyurethane (PU), which together account for the majority of global plastic production [5].

MNPs have been increasingly detected in the human body, raising concerns about their widespread contamination and potential health consequences. In addition, some additives, such as triclosan, bisphenols, phthalates, and brominated flame retardants, can bind to plastic particles, be carried throughout the body, and induce toxicity [10].

Studies indicate that commonly consumed foods may be contaminated with plastic particles, and that inhalation of MNPs suspended in the air can also occur [11,12,13]. Although dermal exposure to MNPs remains poorly documented, recent evidence suggests it may represent a plausible route of human exposure. Potential sources include cosmetics containing microbeads, contact with MNP-contaminated dust, and exposure involving damaged skin, which can facilitate particle penetration [14].

Recent evidence indicates that MNPs can enter the body through multiple exposure routes and subsequently disseminate across various tissues and organs, including the respiratory, digestive, renal, hepatic, cardiovascular, placenta, and central nervous systems, as well as in biological samples such as breast milk, meconium, feces, semen, sputum, and urine [15], reinforcing their capacity for systemic distribution and raising concerns about their potential role in organ dysfunction and fetal development [16,17,18].

The shape and size of MNPs can determine their trajectory within the organism, as smaller, irregular particles are more likely to enter the intracellular environment and cause damage. MP particles smaller than 100 μm and NP particles smaller than 1 μm appear to have a greater probability of crossing organ barriers such as the intestinal, placental, and blood–brain barriers. Larger particles, in turn, tend to be eliminated or remain in the intestinal lumen [15,19,20].

Intestinal translocation of plastic particles is poorly understood but is thought to occur mainly via cellular uptake mechanisms, such as endocytosis, while paracellular transport is unlikely due to strict size limitations (~1.5 nm). Nanoplastics seem to cross the intestinal barrier through both passive diffusion and active endocytic pathways, including clathrin-mediated endocytosis and phagocytosis [21,22]. However, further studies are needed to elucidate the behavior of plastic particles in the human body with greater precision. Although the extent of intestinal translocation remains uncertain, MNPs can already exert biologically relevant effects within the intestine. Ingested MNPs have been shown to alter the composition, functional capacity, and alpha diversity of the gut microbiota, raising concerns regarding subsequent metabolic and inflammatory consequences [23].

A pioneering study observed that, among 22 healthy volunteers, the average concentration of MPs detected in the bloodstream was 1.6 µg/mL, with fragments corresponding to PET, styrene-derived polymers, and poly(methyl methacrylate) [24]. Subsequently, the use of advanced analytical techniques, such as Fourier transform infrared spectroscopy (µ-FTIR), expanded the range of polymers detected in blood, enabling the identification of PP, PE, PS, and PET. The analyses also revealed that the blood concentrations of these particles ranged from 1.84 to 4.65 µg/mL and indicated that higher circulating loads of MPs were associated with alterations in coagulation markers, including fibrinogen, activated partial thromboplastin time (aPTT), and C-reactive protein [25,26].

By directing the investigation of MPs in blood to the placenta, Ragusa et al. (2021) reported, for the first time, the presence of MPs in human placentas obtained from healthy pregnant women using Raman microspectroscopy [18]. Twelve particles with dimensions of 5–10 µm were identified and classified as pigmented PP, a type of thermoplastic polymer [18]. Braun et al. (2021) further corroborated these findings in a pilot study that established a protocol for detecting MPs larger than 50 µm in placental tissue and meconium collected during cesarean deliveries and identified PE, PP, and PS particles [27].

With advances in research on MPs in the maternal context, attention has also turned to human milk, the baby’s first source of food. Studies have detected MPs in approximately 40–75% of human milk samples analyzed, with particles ranging from 2 to 50 µm and composed mainly of PE, PP, PS, PVC, PA, and PU [28,29]. Furthermore, it has been observed that the presence of MPs in breast milk can alter its microbiota, with increased abundances of genera such as *Staphylococcus* and *Streptococcus*, whereas women without MP detection show a higher prevalence of *Enterobacter*, *Escherichia*, *Pseudomonas*, and *Acinetobacter* [30].

In human lung tissue, MPs have been detected in both autopsy analyses and clinical studies. The identified particles ranged from a few to tens of micrometers (5–100 µm), with PP, PE, PS, and PET being the most frequent [31,32,33]. Research has also demonstrated the presence of MPs in human feces, indicating ingestion as an important entry point and the gastrointestinal tract as the route of elimination of these particles. Fecal concentrations vary widely (from 1 to approximately 36 particles/g), and the most commonly detected polymers are PE and PP. A statistically significant relationship was found between higher concentrations of MPs in feces and the use of plastics for storage and food preparation, as well as the consumption of ultra-processed foods [34,35]. More recently, Xu et al. (2025) found that individuals with colorectal cancer have significantly higher amounts of MPs in their feces compared to healthy individuals [36].

Although increasing numbers of studies have reported the presence of MNPs in human tissues, their detection remains technically challenging. Most studies rely on highly specialized analytical techniques, including μFTIR spectroscopy, Raman microspectroscopy, pyrolysis–gas chromatography/mass spectrometry (Py-GC/MS), laser direct infrared imaging, and scanning electron microscopy. These methods are not routinely available in clinical laboratories, and differences in sample preparation, contamination control, polymer identification thresholds, and particle size detection limits contribute to considerable methodological heterogeneity among studies. Therefore, comparisons across studies should be interpreted with caution [37].

## 3. MNPs and Chronic Diseases

Different analytical techniques confirmed the presence of polymers, including PET, PS, and polyacrylonitrile, as well as NPs capable of crossing biological barriers and accumulating in the kidneys, thyroid, and brain [38]. Their presence in these tissues highlights the potential for systemic distribution; however, evidence linking MPs to cardiovascular, metabolic, respiratory, neurological, intestinal, and reproductive disorders as well as tumorigenesis derives primarily from experimental studies investigating mechanisms such as oxidative stress, inflammation, mitochondrial dysfunction, barrier disruption, and cellular senescence [39]. Table 1 summarizes the main findings of in vitro and in vivo studies published between 2021 and 2026 that analyzed the effects of MPs on various tissues, including liver, kidneys, and brain, as well as on cancer.

**Kidney Diseases**—Exposure to MNPs has been associated with potential renal toxicity in experimental in vivo and in vitro models, indicating their capacity for systemic translocation and renal accumulation. In urine samples obtained from six volunteers from different cities in southern Italy, MP fragments of PE, PVC, and PP (4–15 µm) were identified using Raman microspectroscopy [68]. Importantly, MPs and inorganic fragments were identified for the first time in human renal tissue using μ-Raman spectroscopy, providing direct evidence of their presence in the kidney and urine [69].

Compared to the liver and heart, the kidneys exhibit greater deposition of plastic particles due to their high blood flow and highly specialized filtration system. This exacerbates cellular damage, promotes oxidative stress, activates inflammatory responses, induces apoptosis and autophagy, and dysregulates the gut–kidney axis, compromising renal metabolism and contributing to tissue fibrosis [70,71]. Indeed, studies in human kidney-derived cells show that MPs are readily internalized, leading to excessive ROS production, downregulation of antioxidant defenses, mitochondrial dysfunction, metabolic impairment, and activation of inflammatory and autophagic pathways [40,58]. In vivo evidence further indicates that MPs and NPs accumulate in renal tissue, causing structural damage to glomeruli and tubules, immune cell infiltration, oxidative stress, and increased expression of pro-inflammatory cytokines, including TNF-α, IL-6, IL-1β, and MCP-1 [44,49,59].

Recently, researchers evaluated MPs in dialysis solution samples and found predominantly polyethylene, polyvinyl chloride, and ethylene-vinyl acetate fibers, with peritoneal dialysis fluids showing slightly higher concentrations than hemodialysis fluids, highlighting dialysis as a potential route of chronic exposure [72].

It is hypothesized that MPs impair intestinal barrier integrity, increasing the circulation of endotoxins, including lipopolysaccharide (LPS), that activate the complement system, leading to C5a generation and the subsequent recruitment of pro-inflammatory and pro-fibrotic mediators, thereby contributing to kidney injury. However, it is still necessary to expand the human evidence base through longitudinal preclinical studies and refine more sensitive and standardized detection techniques to clarify the biological and clinical relevance of these findings for renal health [73].

**Lung disease**— Some authors indicate that the inhalation of airborne MNPs is a significant route of MNP absorption. Even with the difficulty of fully accessing the lungs due to the mucus lining them, the presence of MNPs in human lung tissues and respiratory fluids has already been demonstrated. Fragments smaller than 10 µm, in particular, can reach the bronchi and even the alveoli, making removal by the body’s respiratory defenses difficult [74,75].

Experimental studies point to several mechanisms responsible for biological damage caused by MP in the lungs. One important mechanism is inflammation induced by the infiltration of inflammatory cells and increased production of cytokines (TNF-α, IL-6), which can be potentiated by the activation of pathways such as TLR2 and NF-κB [60,76].

Studies have suggested a possible association with the development and progression of respiratory diseases, such as Chronic Obstructive Pulmonary Disease (COPD), asthma, and lung cancer. Regarding COPD, MPs can trigger significant airway inflammation characterized by oxidative stress and alveolar destruction, thereby increasing the likelihood of ferroptosis and cellular injury [53,77]. In relation to asthma, this accentuated inflammation is accompanied by eosinophil infiltration and increased mucus synthesis and reactivity, leading to morphological changes in airway tissues [77,78]. Regarding lung cancer, direct evidence in humans is still incipient, but recent studies reveal hypotheses about the collaboration of MPs in pulmonary carcinogenesis through inflammation and oxidative stress [79,80]. Although recent evidence consistently indicates potential risks of plastic microparticles (MPs) to lung health, more robust epidemiological studies are still needed to determine whether a causal relationship exists between environmental exposure to plastic particles and the occurrence of lung diseases in humans.

**Neurological disease**—MPs and NPs can also interact with the central nervous system, highlighting a new and relevant area of concern for neurological health. Experimental evidence indicates that NPs can act on the central nervous system through different complementary pathways. Experimental studies consistently show that MNPs accumulate in the brain and disrupt the blood–brain barrier, triggering neuroinflammation, oxidative stress, and synaptic dysfunction, ultimately impairing cognition. At the molecular level, NPs promote the aggregation of neurodegeneration-related proteins (e.g., α-synuclein and β-amyloid) and impair neuronal function. In parallel, gut dysbiosis and increased intestinal permeability appear to contribute to these effects via the gut–brain axis, reinforcing the role of systemic inflammation in neurotoxicity [45,51,62].

Recently, a human study provided the first evidence of MPs in cerebrospinal fluid (CSF) and explored the association between MPs and biomarkers related to Alzheimer’s disease (AD) [67]. PE and PVC concentrations were significantly higher in the CSF of amyloid-positive individuals; meanwhile, PE levels were inversely correlated with CSF Aβ42 concentrations and Mini-Mental State Examination (MMSE) scores, and positively associated with cognitive decline over the course of a year. Although these findings do not establish causality, they suggest a potential relationship between microplastic accumulation in the CSF and AD-related changes [67].

Taken together, the available experimental and clinical evidence suggests that exposure to MNPs may act as a potentiating factor in neurodegenerative processes. These effects appear to occur through both direct mechanisms, such as interaction with proteins implicated in diseases like Parkinson’s and Alzheimer’s, and indirect mechanisms involving intestinal dysfunction, systemic inflammation, and cellular impairment. Although the mechanisms are still being consolidated, the evidence reinforces the need for in-depth investigations into the neurological impact of these particles amid increasing environmental exposure.

**Liver disease**—The interaction between MNPs and the liver has emerged as one of the most important pathophysiological processes in current environmental toxicology. MNPs can be absorbed in the intestine and subsequently reach the liver via the portal vein, causing liver inflammation, metabolic disorders, and oxidative stress in liver cells, which can result in inflammation and cell death [81,82]. Also, a disruption of the intestinal barrier caused by MNPs allows endotoxins to enter the bloodstream, triggering inflammatory responses in the liver. Exposure to MNPs is associated with elevated levels of triglycerides and cholesterol in this organ, contributing to conditions such as non-alcoholic fatty liver disease (NAFLD) [83]. Indeed, research with mice and zebrafish has demonstrated that MPs contribute to NAFLD symptoms, including lipid accumulation and liver inflammation, highlighting the relevance of these findings for understanding the risks to human health [41,84,85]. Researchers have also shown that MPs can positively regulate genes associated with lipid accumulation [55]. In addition, the correlation between MPs and human hepatocellular carcinoma has been observed, emphasizing the need for further research [54].

**Cancer Development**—MNPs are increasingly recognized as significant contributors to cancer development through mechanisms involving chronic inflammation and oxidative stress, leading to cellular damage and DNA mutations, which are crucial in cancer progression. These tiny plastic particles can trigger pro-inflammatory responses that sustain chronic inflammation, creating a microenvironment conducive to tumor initiation [86,87]. Indeed, recent research has demonstrated that MNP particles can activate signaling pathways associated with tumor development, such as MAPK/ERK and PI3K/AKT, leading to increased DNA damage and enhanced cell survival [54,55].

A study collected human colorectal cancer (CRC) tumor tissues and blood samples and analyzed them for MPs. In parallel, mice chronically exposed to these MPs were used to assess the effects on the gut microbiota and the efficacy of CRC immunotherapy. MPs induced gut microbiota dysbiosis, impaired antitumor immunity, and promoted colorectal cancer progression and resistance to immunotherapy [88].

Another critical point is the transport of carcinogenic additives, such as bisphenol A (BPA), alpha-zeranol (αZAL), and di(2-ethylhexyl) phthalate (DEHP), which frequently adhere to MPs and act as endocrine disruptors, modulating hormonal pathways involved in cancers such as breast cancer. Studies show that these disruptors carried by MPs can stimulate breast cell proliferation, increase hormone receptor expression, and promote genomic instability [89].

Therefore, exposure to MPs can act at multiple levels (inflammation, oxidative damage, genotoxicity, dysbiosis, metabolism, and cell signaling), creating a biologically plausible scenario for tumor initiation and promotion in exposed tissues.

**Cardiovascular disease**—Experimental and clinical evidence suggests that MNPs may contribute to cardiovascular disease (CVD) through multiple mechanisms, including oxidative stress, endothelial dysfunction, chronic inflammation, metabolic dysregulation, and thrombogenic processes [66]. Preclinical studies have demonstrated that ingestion of polystyrene MPs induces metabolic alterations associated with CVD. In mouse models, chronic exposure to MPs promotes adiposity, obesity, and insulin resistance through gut microbiota remodeling and dysregulated lipid metabolism, which are recognized as determinants of cardiometabolic diseases and atherosclerosis [47,50]. Experimental evidence also shows that MPs can impair endothelial function by increasing ROS production, triggering mitochondrial dysfunction, and activating inflammatory pathways via the ROS-driven NF-κB-NLRP3-GSDMD and AMPK-PGC-1α axes, as well as intestinal barrier dysfunction via the NF-κB/NLRP3/IL-1β/MCLK pathway [90,91,92].

Recent evidence from human induced pluripotent stem cell-derived cardiomyocytes (hiPSC-CMs) further supports the cardiotoxic potential of MPs. Chronic exposure to low-dose polystyrene MNPs reduced cell viability, impaired contractility and calcium homeostasis, exacerbated cardiomyocyte hypertrophy, and promoted mitochondrial dysfunction and oxidative stress, highlighting direct cellular mechanisms that may contribute to cardiovascular injury [93]. MNPs can also interfere with coagulation pathways and platelet activation, potentially increasing the risk of thrombotic events [94].

In a prospective multicenter observational study of patients undergoing carotid endarterectomy, polyethylene and polyvinyl chloride were detected in carotid atherosclerotic plaques using advanced analytical techniques. Importantly, patients with detectable MNPs within the atheroma had a significantly higher risk of major adverse cardiovascular events, including myocardial infarction, stroke, and all-cause mortality, during a mean follow-up of 34 months [66].

MNPs were detected more frequently and at higher concentrations in the coronary blood of patients with ST-segment elevation myocardial infarction (STEMI) than in those with chronic coronary syndromes or normal coronary arteries. Furthermore, MNP detection was associated with higher PM2.5 exposure, suggesting that environmental exposure to MNPs may be linked to established cardiovascular risk factors and a more pronounced inflammatory profile. Although the cross-sectional design precludes inferring causality, these findings reinforce the biological plausibility of a relationship between MNP exposure and cardiovascular disease [95].

Taken together, the available evidence indicates that MNPs may influence CVD through a complex network of metabolic, inflammatory, and vascular mechanisms. Although human data remain limited, the growing body of experimental research highlights the need for further epidemiological studies to clarify the clinical relevance of chronic exposure to MPs.

**Figure 1:**
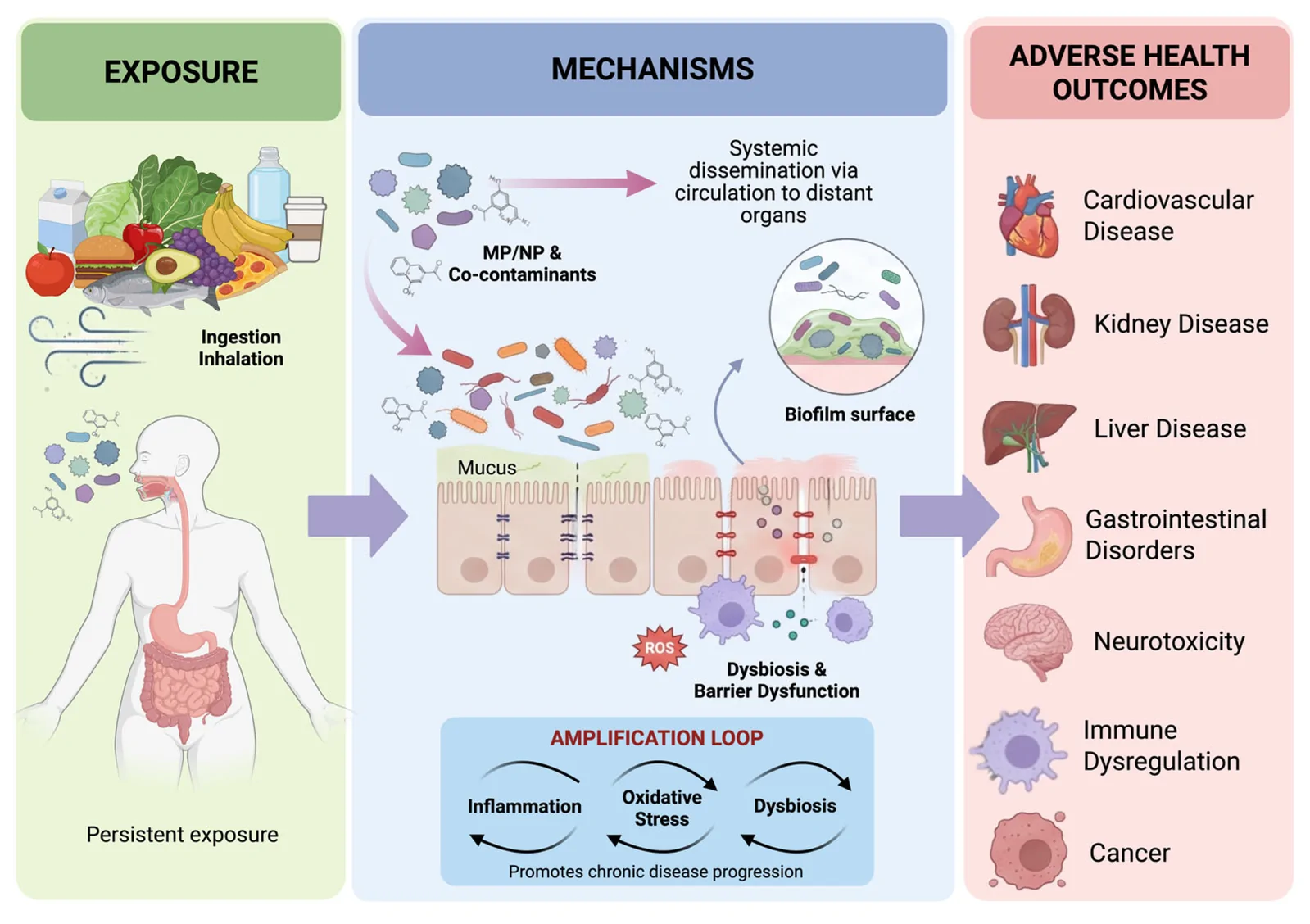
Proposed pathway linking MNP exposure to chronic inflammation and adverse health outcomes. Created by BioRender.com.

Figure 1 summarizes the proposed biological pathway linking chronic exposure to micro- and nanoplastics (MNPs) with adverse health outcomes. MNPs may enter the body primarily through ingestion and inhalation and, depending on their size, physicochemical properties, and associated contaminants, may interact with the intestinal and respiratory barriers and potentially reach the systemic circulation. Their biological effects may involve direct cellular interactions, oxidative stress, immune activation, barrier dysfunction, and interactions with the gut microbiota. In the intestine, dysbiosis and disruption of the epithelial barrier may further promote the translocation of microbial-derived products and amplify inflammatory responses. In parallel, MNP-associated biofilms and co-contaminants may contribute to additional biological effects. The resulting interplay among inflammation, oxidative stress, and microbial dysbiosis may establish a self-reinforcing cycle that contributes to chronic tissue injury and disease progression across multiple organ systems.

Beyond their direct toxic effects on individual organs, MNPs may also influence host physiology indirectly by disrupting the intestinal ecosystem. Increasing evidence indicates that gut microbial dysbiosis and impaired intestinal homeostasis could contribute to the systemic consequences of chronic MP exposure, providing an additional mechanistic framework for understanding their association with chronic diseases.

## 4. MNPs as Disruptors of Gut Microbial and Intestinal Homeostasis

Experimental studies indicate that even short-term exposure to MPs can lead to significant alterations in gut microbiota composition and diversity [96]. Complementarily, in vitro models have demonstrated that exposure to PET reshapes the colonic microbial community by directly interacting with resident microorganisms, favoring microbial aggregation and the formation of biofilm-like structures. Certain bacterial species commonly found in the human intestinal tract, such as *Escherichia coli*, have demonstrated the ability to adhere to and form biofilm-like structures on plastic surfaces, which may facilitate the colonization and protection of microbial communities [97].

Consequently, MPs can act as ecological niches for microbial colonization and as vectors, facilitating the transport of microorganisms, antibiotic resistance determinants, and adsorbed environmental contaminants [98]. Furthermore, these plastic materials can contribute to gut microbiota dysbiosis and the generation of systemic inflammatory responses, as they can also adsorb a wide range of environmental contaminants, such as heavy metals and persistent organic pollutants, thereby exacerbating issues like oxidative stress and inflammatory signaling that intensify intestinal dysfunction [99].

The deposition of MNPs in the lumen can compromise the integrity of the epithelium and mucus layer, leading to wear, reduced mucus thickness, and altered microvillar morphology. Experimental studies indicate that exposure of epithelium to MNPs, particularly PS, increases reactive oxygen species (ROS) production, thereby activating pro-inflammatory signaling pathways, including NF-κB, MAPK, and the NLRP3 inflammasome. This activation stimulates the release of cytokines, including tumor necrosis factor-α (TNF-α), interleukin-1β (IL-1β), and interleukin-6 (IL-6), thereby establishing a local pro-oxidative and pro-inflammatory milieu within the intestinal environment [91].

Inflammatory activation leads to loss of tight junction proteins (occludin, claudins, and ZO-1), increased intestinal permeability, and impaired intestinal barrier integrity. Particle size and morphology critically influence cellular adhesion and internalization, with smaller particles (particularly NPs) being more readily taken up by intestinal epithelial cells and eliciting more pronounced inflammatory responses. Particle shape, including spheres, fibers, and irregular fragments, further affects intestinal toxicity through differences in surface charge and the presence of associated chemical additives. In addition, exposure dose and duration, as well as the organism’s age, substantially modulate the inflammatory outcome, with prolonged or repeated exposures leading to more persistent inflammatory states [100,101]. Host-related factors, including immune status, dietary patterns, gut microbiota composition, and pre-existing intestinal conditions, may further increase susceptibility to the pro-inflammatory effects of MP exposure [99]. Table 2 presents studies involving MNPs and gut microbial composition and intestinal barrier function.

A recent systematic review reported that experimental animals exposed to polystyrene exhibit gut dysbiosis, characterized by reduced diversity, depletion of beneficial taxa such as *Lactobacillus*, *Bifidobacterium*, and *Ruminococcaceae*, and enrichment of pro-inflammatory bacteria, including *Proteobacteria*, *Helicobacter*, and *Staphylococcus* [119].

**Figure 2:**
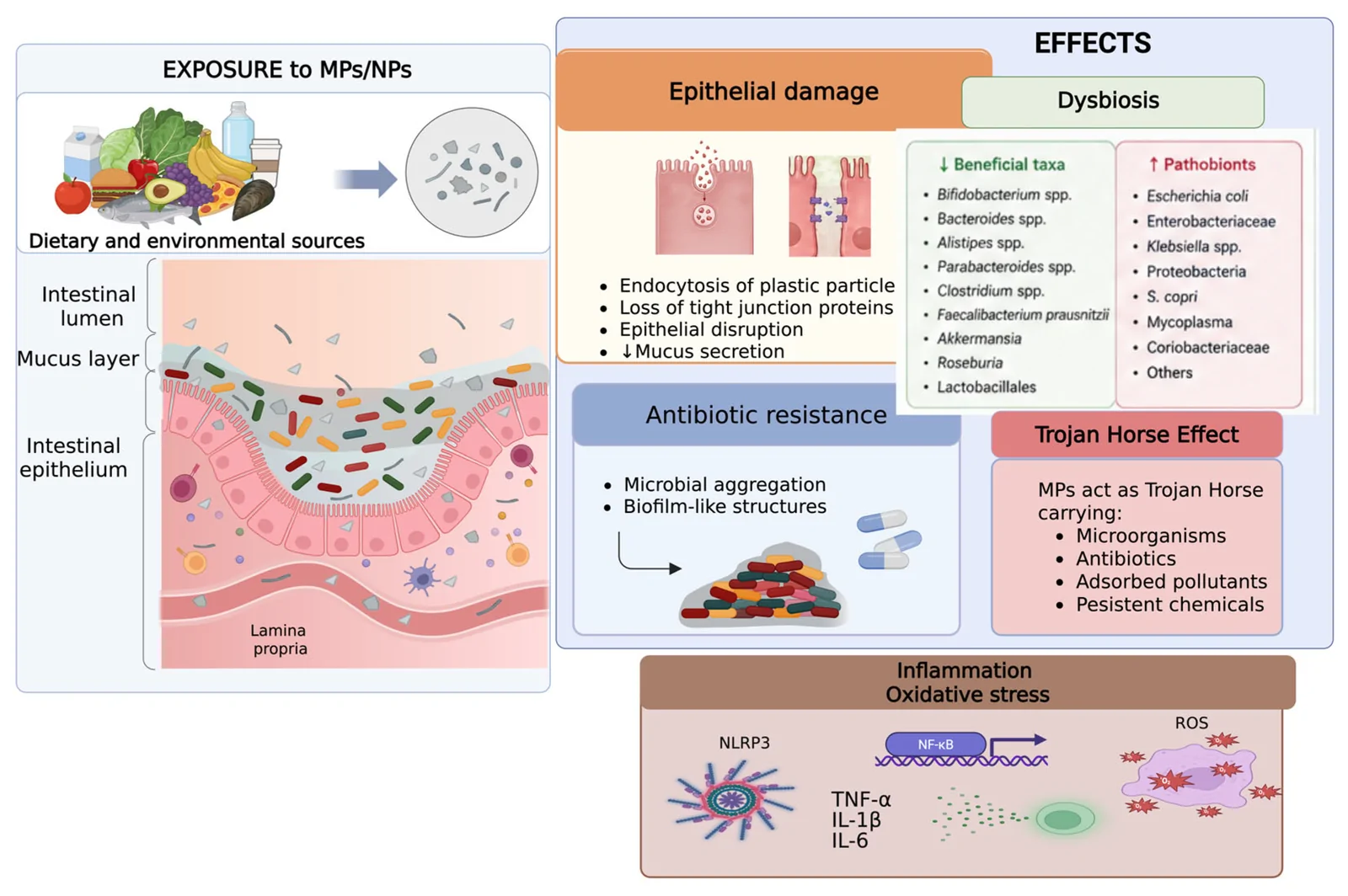
Proposed mechanisms linking microplastic and nanoplastic exposure to intestinal dysfunction. Microplastics and nanoplastics (MNPs) derived from dietary and environmental sources can interact with the intestinal ecosystem, including the mucus layer, gut microbiota, and epithelial barrier. Experimental evidence suggests that MNPs may induce gut dysbiosis, disrupt epithelial integrity by causing loss of tight junction proteins and altering the mucus barrier, and increase intestinal permeability. Furthermore, MNPs may facilitate microbial aggregation and biofilm formation, potentially favoring antimicrobial resistance. These disturbances can activate inflammatory and oxidative stress pathways, including NLRP3 inflammasome activation, NF-κB signaling, increased production of cytokines (TNF-α, IL-1β, and IL-6), and reactive oxygen species generation. Together, these effects may compromise intestinal homeostasis and contribute to local and systemic inflammation. Created by BioRender.com.

Compromised intestinal barrier function facilitates the translocation of MNPs and microbial products into the systemic circulation. Together with gut microbiota dysbiosis, these alterations promote sustained inflammatory responses and establish a self-reinforcing cycle of intestinal dysfunction. In this context, exposure to MNPs can remodel the gut microbial ecosystem, contributing to the development of inflammatory bowel diseases, chronic diseases, and even neurological disorders [99]. Despite growing experimental evidence, critical knowledge gaps remain, as human epidemiological data are limited and the consequences of long-term, low-dose exposure are still poorly characterized [100]. The proposed mechanisms linking MP and NP exposure to intestinal dysfunction are summarized in Figure 2.

## 5. Research Gaps and Future Perspectives

A recent review analyzing methods for extracting and quantifying MNPs in biological tissues outlined a range of protocols, including reagent-based and chemical digestion methods, as well as distinct analytical techniques. This diversity hinders the reproducibility and comparison of study results, making it difficult to obtain a more reliable estimate of the actual number of MPs in organs and tissues [37]. Therefore, standardized, validated protocols focused on biological samples with clear definitions of size ranges and MP formats, comparable analyses, digestion techniques adapted to the samples, and minimum quality parameters are becoming increasingly necessary. This standardization would enable the integration of studies into meta-analyses, yielding more robust results and, consequently, advancing understanding of the potential risks of MPs to human health.

Another gap in research on MNPs’ influence on the microbiota and health is the scarcity of longitudinal human studies, as most evidence comes from cross-sectional studies, in vitro digestion simulation models, or animal studies. This context highlights the urgency of prospective cohorts capable of evaluating MNP exposure over time and its correlation with changes in the microbiota, inflammation, and disease emergence [23].

The multiomics approach encompasses strategies such as epigenomics, transcriptomics, and lipidomics, which are important for understanding numerous diseases and dysbiosis resulting from the ingestion of contaminated food. These techniques favor the understanding of the mechanisms of alteration of the intestinal microbiota due to the influence of MPs, which requires both the inclusion of taxonomic (metagenomics), metabolic (metabolomics/lipidomics), and functional (proteomics) data, as well as a detailed description of human exposure to these particles (exposomics). Thus, multiomics approaches that address the issues raised in the previous paragraphs can identify compromised metabolic and immunological pathways and propose more effective solutions to mitigate damage to the microbiome [120].

## 6. Conclusions

MNPs are emerging as novel environmental stressors that can alter the gut microbiota, promoting inflammation and various diseases, including cancer, lung, cardiovascular, and even neurological problems. More research is essential to clarify dose–response relationships, identify vulnerable populations, and guide public health policies to reduce plastic exposure.

## Figures and Tables

**Table 1:** In vitro and in vivo studies analyzing the effects of MNPs on various tissues and cancer.

Authors (Year).	Study Model	Key Outcomes
In vitro		
Goodman; Hua; Sang (2022) [40]	Human Embryonic Kidney (HEK293) cells using polystyrene (1 µm)	↑ ROS; ↓ antioxidant enzymes (SOD2, CAT); metabolic disruption; structural and functional cellular damage; metabolic toxicity
Cheng, Wei et al. (2022) [41]	Liver organoids with polystyrene MPs (~1–10 μm)	Hepatotoxicity; oxidative stress; mitochondrial dysfunction
Gou et al. (2024) [42]	Human neuroblastoma (SHSY-5Y) cells with polystyrene MPs	↑ aggregation of β-amyloid proteins (Aβ_40_ and Aβ_42_); damage to cell membranes; neurodegeneration
Kim; Park; Oh (2026) [43]	Normal and Cancerous human lung cells with polystyrene NPs (50, 100, 300, and 1000 nm)	↑ sensitivity due to ↓ in the antioxidant enzyme SOD1 (redox imbalance) in cancer cells; ↑ cell migration (cytoskeletal remodeling) without loss of viability in normal cells
Animal models		
Gao, Qi et al. (2022) [44]	Mice with PET NPs (200 nm)	PET NPs were distributed throughout the liver, spleen, lungs, and kidneys; The NPs were rarely detected in peripheral organs, suggesting limited passage across the intestinal barrier.
Jin, Haibo et al. (2022) [45]	Mice with polystyrene MPs	↑ IL-6, IL-1β, TNF-α, MCP-1 and CXCL10; lipid damage; structural neuronal damage and impaired memory and learning performance
Zhang, Yue et al. (2022) [46]	Chicken with polystyrene MPs (~5 μm)	NF-κB/NLRP3 activation; AMPK-PGC-1α inhibition; cardiotoxicity and inflammation
Huang, Dingjie et al. (2022) [47]	Mice with polystyrene MPs (~5 μm)	Gut dysbiosis; systemic inflammation; insulin resistance
Shen et al. (2023) [48]	Mice with polystyrene MPs (environmental concentrations)	Mitochondrial dysfunction; altered oxidative phosphorylation and thermogenesis pathways; structural and functional renal damage; transcriptomic alterations
Xiong et al. (2023) [49]	Mice with MPs (80 nm, 0.5 µm, 5 µm)	Chronic kidney injury; macrophage infiltration; circadian gene disruption; oxidative stress; apoptosis; immune dysregulation; ↑mRNA expression of MCP-1 and IL-1β
Huang, Haipeng et al. (2023) [50]	Mice with MPs (~1–10 μm)	Dysbiosis; ↑ adiposity
Liu et al. (2023) [51]	Mice with NPs	↑ fibrillar aggregation of the α-synuclein protein; ↑ cellular damage; lysosomal dysfunction; ↑ neurodegenerative processes
Pan et al. (2024) [52]	Mice with polystyrene MPs	Tubular cell senescence; Klotho/Wnt/β-catenin activation; EMT; renal fibrosis; extracellular matrix accumulation
Yang et al. (2024) [53]	Mice with polystyrene NPs (40 nm)	Local and systemic toxicity; inflammation; lung injury; ↑ ROS; ↓ CAT, SOD and GSH-Px; ↑ IL-6, MCP-1 and TNF-α
Huang, Haipeng et al. (2024) [54]	Mice using MPs (~1–10 μm)	↑ liver tumorigenesis
Chiu et al. (2025) [55]	Mouse with polystyrene MPs (~5 μm)	NR4A1–AMPK pathway dysregulation; ↑ hepatic lipids
Huang, Haipeng et al. (2025) [56]	Mice with MPs	↑ phagocytosis of MPs by monocytes/macrophages; ↓ cerebral perfusion; local hypoxia; neuronal damage; capillary thrombosis
Choi et al. (2025) [57]	Mice using ovalbumin-induced acute asthma model using polystyrene MPs (1–5 µm)	Induced inflammation, pro-inflammatory cytokines (TNF-α), epithelial alarmins (IL-25, IL-33), and polarization of M1 macrophages in healthy lungs; ↓ eosinophilic infiltration and Th2 cytokines (IL-5, IL-13) in asthmatic lungs
In vitro and in vivo		
Wang, Yung Li et al. (2021) [58]	Human proximal tubule (HK-2) cells; mice using polystyrene	↑ MPs; ↑ cPLA2α and COX-1; renal inflammation; mitochondrial dysfunction; ER stress; ↑ ROS; autophagy
Meng et al. (2022) [59]	Mice using polystyrene NPs (50 nm); MPs (300 nm, 600 nm, 4 µm)	Oxidative stress; inflammatory signaling (TNF-α, IL-6, MCP-1); renal structural damage; impaired function; weight loss; ↑ mortality
Cao et al. (2023) [60]	Human lung adenocarcinoma (A549 cells) Mice using polystyrene MPs (small: 1–5 μm and large: 10–20 μm)	Inflammation, oxidative stress, apoptosis, and fibrosis in the lungs; ↑ SOD and ROS; ↑ apoptosis rate; ↑ fibrosis markers; NF-κB pathway activation
Jin, Wenhua et al. (2024) [61]	Human lung epithelial cells; mouse lungs using MPs	↑ ROS; pro-senescent pulmonary response
Jeong et al. (2024) [62]	Nematode *Caenorhabditis elegans*; human cell models of Parkinson’s using polystyrene NPs (25 nm)	They inhibit growth and movement in *C. elegans*; induce a “leaky gut” condition and penetrate extraintestinal tissues; exacerbate Parkinson’s symptoms, including dopaminergic neuronal degeneration and ↑ α-synuclein aggregates in both models
Yuan et al. (2025) [63]	Ovarian cancer cells; mice using polystyrene NPs (50 nm)	Promoting tumor progression; ↑ cell proliferation via CDK4/6-dependent signaling; cell internalization predominantly by clathrin-mediated endocytosis
Baek et al. (2026) [64]	Mice on a liquid diet with ethanol; intestinal cells Caco-2 and HT-29 with polystyrene MPs (2.16 µm)	Disruption of the gut–liver axis; damage to tight junctions; ↓ ZO-1, occludin, and F-actin, facilitating the translocation of plastics to the lamina propria and accumulation in the liver
Shanmugiah et al. (2026) [65]	2D bronchial epithelial organoids and A549 cells; mice—inhalation for 6 and 12 weeks) using polystyrene (0.25 µm and 20 nm)	Size-dependent pulmonary toxicity: smaller PS particles were more pathogenic; activation of the EGFR-dependent signaling pathway (MAPK) involving AREG and MAP3K13; ↓ lung volume; ↓ exercise capacity; induction of cancer markers (PD-L1, CD44)
Human Studies		
Marfella et al. (2024) [66]	Clinical (human carotid plaques) with MPs and NPs	Plaque inflammation; instability; ↑ cardiovascular events
He et al. (2025) [67]	Human observational	↓ Aβ42 in CSF; cognitive decline; β-amyloid deposition; progressive neuronal damage

Abbreviations: ER: endoplasmic reticulum; ROS: reactive oxygen species; EMT: epithelial–mesenchymal transition; CSF: cerebrospinal fluid; EGFR: epidermal growth factor receptor; MAPK: mitogen-activated protein kinases; AREG: amphiregulin; PET: polyethylene terephthalate.

**Table 2:** Experimental and clinical evidence on the effects of MNPs on gut microbial composition, intestinal barrier function, and intestinal homeostasis.

Authors (Year)	Model/MNP Type	Main Findings
In vitro studies		
Souza-Silva et al. (2022) [102]	Mechanistic analysis with MPs	Expansion of Proteobacteria (pro-inflammatory taxa) Endotoxemia; immune activation, ↑ LPS-related inflammatory tone
Tamargo et al. (2022) [97]	Metagenomics with MPs	Dysbiosis; ↓ beneficial taxa (↓ Bifidobacterium spp., Bacteroides, Alistipes, Parabacteroides, and *Clostridium* spp.), barrier dysfunction pro-inflammatory shift
Donkers et al. (2022) [103]	Human intestinal and pulmonary models with nylon fibers; HDPE fragments; MNPs	↓ tissue function; ↑ IL-6
Ding et al. (2024) [104]	(Caco-2 cells) using polystyrene-MPs ± nonylphenol	ROS; MAPK activation; apoptosis
Brouwer et al. (2025) [105]	Human iPSC-derived intestinal epithelium using PET-TiO_2_, PVC, PP-talc, polyamide	Barrier disruption; ↑ IL-6 and IL-8 oxidative stress
Animal models		
Jin, Yuanxiang et al. (2019) [106]	Mice, oral exposure with polystyrene (pure and fluorescent) for 6 weeks	Intestinal barrier disruption; metabolic imbalance; ↓ mucus secretion
Chen, Xuanwei et al. (2024) [107]	Murine model, oral exposure with polystyrene NPs	NF-κB/NLRP3 activation; inflammatory cell recruitment; ↓ tight junction proteins; ↑ permeability intestinal and hepatic inflammation
Zeng et al. (2024) [91]	Mice, oral exposure with polystyrene MPs	NF-κB/NLRP3 inflammasome activation; oxidative stress; ↓ junctional proteins; increased permeability
Liang et al. (2024) [108]	Murine model, oral exposure with polystyrene NPs	Dysbiosis (↑ Mycoplasma, Coriobacteriaceae, *Mesorhizobium* and *Lwoffii*); ↑ IL-17C; brain damage
Sung et al. (2025) [109]	Mice, oral exposure with polyethylene MPs	Tight junction disruption (↓ ZO-1 and occluding)
Afridi et al. (2026) [110]	Gut microbiome of zebrafish with polyethylene-MPs + glyphosate	Direct stressors in the gut microbiome: ↓ α-diversity; ↓ *A. veronii*; ↑ *A. hydrophila*
Wu et al. (2026) [111]	Honeybees (*Apis mellifera*) with polystyrene-MP + glyphosate	↓ α-diversity; ↓ *Gilliamella*, *Bifidobacterium*, *Snodgrassella*, *Apilactobacillus* ↑ mortality; ↑ midgut damage; ↑ dysbiosis; ↓ serotonergic synapse-related genes
Zhai et al. (2026) [112]	Sulfate sodium (DSS)-induced colitis in mice with polystyrene microspheres	↓ *Lachnospiraceae_NK4A136_group* ↓ butyrate levels; ↓ PPARγ signaling; ↑ colitis
Wang, Qiaoling et al. (2026) [113]	Female mice exposed with polyethylene for 4 weeks	Dysbiosis (↓ *Akkermansia* abundance) ↓ myristic acid, phenylacetylglycine, ↓ GLP-1, PYY, AMH, testosterone
Qin et al. (2026) [114]	Broiler chickens = Control group and MPs intake group (300 mg/kg)	Dysbiosis (↓ *Actinobacteriota*, *Desulfobacterota*, *Acidobacteriota*; ↑ Firmicutes), 11 bacterial genera disappeared, ↓ T-AOC, GSH-Px and SOD levels; ↑ MDA
Human Studies		
Ke et al. (2023) [115]	Human observational (children)—MPs (fecal)	Dysbiosis (↓Lactobacillales, Rikenellaceae, Alistipes, and Streptococcus), ↓ diversity; altered probiotic taxa
Gao, Bei et al. (2025) [116]	Humans (fecal and blood samples) and mice (oral exposure for 14 weeks)—PVC, PE, PP, PS, and PA66 (in human blood); PS (in mice)	Dysbiosis (↑ *Enterobacteriaceae* and *Escherichia coli*, ↓ *Faecalibacterium prausnitzii*); ↑ microbial invasion capacity in tissues; PS altered the gut microbiota of mice; deregulation of the quorum sensing system; ↑ virulence genes related to invasion
Ma et al. (2025) [117]	Humans (Chinese elderly)—MPs (fecal sample)	Changes in β-diversity (↑ *Klebsiella*, *Escherichia-Shigella*, and Enterobacter; ↓ *Blautia*, *Monoglobus*, and *Roseburia*); ↑ MDI; oxidative stress and inflammation via LPS production; alterations in metabolites
Song et al. (2026) [118]	Humans (fecal sample) PE, PVC, PS, PP, PET and PA66	Dysbiosis (↑ *S. copri*, *Escherichia coli*, and *Bacteriophage* sp.), structural impact of PS on the microbiota; change in α and β-diversity

Abbreviations: LPS: lipopolysaccharide; HDPE: high-density polyethylene; ZO-1: zonula occludens-1; PET-TiO_2_: polyethylene terephthalate with titanium dioxide; PP-talc: polypropylene–talc; DSS: dextran sulfate sodium; PPARγ: peroxisome proliferator-activated receptor γ; GLP-1: glucagon-like peptide-1; PYY: peptide YY; AMH: anti-Müllerian hormone; T-AOC: total antioxidant capacity; PA66: polyamide 66; MDI: microbial dysbiosis index; ROS: reactive oxygen species; GSH-Px: glutathione peroxidase; SOD: superoxide dismutase.

## Data Availability

No new data were created or analyzed in this study. Data sharing is not applicable to this article.

## Abbreviations

The following abbreviations are used in this manuscript:

MPs.	Microplastics
PA	Polyamide
PVC	Polyvinyl chloride
PET	Polyethylene terephthalate
PU	Polyurethane
LDPE	Low-density polyethylene
PET-TiO_2_	Polyethylene terephthalate with titanium dioxide
aPTT	Activated partial thromboplastin time
ROS	Reactive oxygen species
TNF-α	Tumor necrosis factor-α
IL-6	Interleukin-6
NLRP3	NLRP3 inflammasome
MAPK	Mitogen-activated protein kinases
SOD	Superoxide dismutase
GSH-Px	Glutathione peroxidase
ZO-1	Zonula occludens-1
MDI	Microbial dysbiosis index
COPD	Chronic obstructive pulmonary disease
CSF	Cerebrospinal fluid
BPA	Bisphenol A
GLP-1	Glucagon-like peptide-1
AMH	Anti-Müllerian hormone
AMPK	AMP-activated protein kinase
EMT	Epithelial–mesenchymal transition
Aβ42	Beta-amyloid 42
μ-FTIR	Fourier-transform infrared spectroscopy
NPs	Nanoplastics
PE	Polyethylene
PP	Polypropylene
PS	Polystyrene
HDPE	High-density polyethylene
PA66	Polyamide 66
PP-talc	Polypropylene–talc
PCBs	Polychlorinated biphenyls
MNPs	Micro- and nanoplastics
IL-1β	Interleukin-1β
NF-κB	Nuclear factor kappa B
LPS	Lipopolysaccharide
MDA	Malondialdehyde
CAT	Catalase
T-AOC	Total antioxidant capacity
SCFA	Short-chain fatty acids
DSS	Dextran sulfate sodium
CVD	Cardiovascular disease
NAFLD	Non-alcoholic fatty liver disease
PPARγ	Peroxisome proliferator-activated receptor γ
PYY	Peptide YY
EGFR	Epidermal growth factor receptor
ER	Endoplasmic reticulum
AREG	Amphiregulin
Aβ40	Beta-amyloid 40
